# An observational study to determine the relationship between cough frequency and markers of inflammation in severe asthma

**DOI:** 10.1183/13993003.03205-2021

**Published:** 2022-12-08

**Authors:** Joshua Holmes, Lorcan P.A. McGarvey, Surinder S. Birring, Hannah Fletcher, Liam G. Heaney

**Affiliations:** 1 Wellcome-Wolfson Institute for Experimental Medicine, Belfast, UK; 2 Centre for Human and Applied Physiological Sciences, School of Basic and Medical Biosciences, Faculty of Life Sciences and Medicine, King's College London, London, UK

## Abstract

**Background:**

The relationship between objectively measured cough and type 2 (T2) biomarkers and other measures of asthma control and severity is poorly understood. The objective of this study was to assess the relationship between objective and subjective cough measurement tools and clinical biomarkers of asthma.

**Methods:**

Patients with severe asthma and mild-to-moderate asthma completed validated asthma and cough-related measurement tools (including ambulatory cough monitoring) and measurement of spirometry and T2 biomarkers (exhaled nitric oxide fraction (*F*_ENO_) and peripheral blood eosinophil count). Patients were classified according to T2 status based on T2-low (*F*_ENO_ <20 ppb and peripheral blood eosinophils <150 cells·µL^−1^), T2-intermediate (*F*_ENO_ ≥20 ppb or peripheral blood eosinophils ≥150 cells·µL^−1^) or T2-high (*F*_ENO_ ≥20 ppb and peripheral blood eosinophils ≥150 cells·µL^−1^).

**Results:**

61 patients completed the study measurements (42 severe asthma and 19 mild-to-moderate asthma). Patients with severe asthma had higher rates of cough than those with mild-to-moderate asthma in terms of total 24-h cough counts (geometric mean±sd 170.3±2.7 *versus* 60.8±4.1; p=0.002) and cough frequency (geometric mean±sd 7.1±2.7 *versus* 2.5±4.1 coughs·h^−1^; p=0.002). T2-low patients with severe asthma had significantly lower 24-h cough frequency compared with T2-intermediate and T2-high patients.

**Conclusions:**

In patients with low biomarkers of T2 inflammation, cough frequency measurements were not elevated, suggesting that the mechanism for cough in asthma is underlying T2 eosinophilic inflammation and the logical first step for treating cough in asthma may be to achieve adequate suppression of T2 inflammation with currently available therapies.

## Abstract 2

**Although significant cough burden can be identified in patients with severe asthma, this may not be present in patients with low levels of T2 inflammatory biomarkers** https://bit.ly/3yqSKVg

## Introduction

It is estimated that asthma affects 1–18% of the population in different countries [1]. While the majority of patients with asthma have mild-to-moderate disease, there remains a significant unmet need with up to 20% of patients failing to achieve acceptable levels of disease control despite receiving treatment with high-dose anti-inflammatory and bronchodilator therapy; these patients are referred to as having “difficult-to-treat asthma” [1, 2]. One attractive approach to managing this difficult patient group is to break them down into separate component parts, also known as “treatable traits”, which can be clinically identified and addressed [3]. This approach essentially deconstructs airway disease into multiple clinical factors, which can include airways inflammation, airflow obstruction, bronchial hyperresponsiveness and cough reflex hypersensitivity [4].

Cough is a common symptom within asthma that has been shown to be very important to patients [5] and troublesome cough has been associated with poor asthma control [6]. However, cough as a symptom is not captured well in commonly used patient-reported outcome (PRO) measures, *e.g.* cough is not included in the Asthma Control Questionnaire (ACQ) [7] and the Asthma Control Test asks about cough in the context of a broad collection of symptoms (wheezing, coughing, shortness of breath, chest tightness or pain) [8]. In keeping with this, previous work has shown that objective assessment of cough, using ambulatory cough monitoring, has a limited relationship with these measures of asthma control [9] and that cough quality of life measures (such as the Leicester Cough Questionnaire (LCQ)) are only moderately correlated with ACQ and Asthma Quality of Life Questionnaire (AQLQ) scores, and not with eosinophilic airways inflammation in sputum [10]. However, there is limited knowledge of how objective cough measurements relate to biomarkers of type 2 (T2) airways inflammation. Therefore, the aim of this study was to explore the relationships between objective cough frequency, cough quality of life, asthma control and T2 biomarkers in severe asthma.

## Methods

### Study design and participants

This was a single-centre prospective observational study. Patients were recruited between October 2017 and September 2019. Full inclusion and exclusion criteria are available in the supplementary material. In brief, patients with severe asthma who were currently stable were recruited from a specialist severe asthma service (Belfast City Hospital, Belfast, UK); all were on Global Initiative for Asthma (GINA) Step 4/5 treatment and had been through a systematic full diagnostic evaluation protocol to confirm the diagnosis of asthma [11]. A comparison cohort of mild-to-moderate asthmatic patients, defined as being on treatment Step 2/3 as per GINA guidelines and stable at the time of recruitment, were recruited from local general practice surgeries. None of the patients in either group were selected on the basis of the presence or absence of cough as a predominant clinical symptom.

This study received ethical approval from the Office of Research Ethics Committees, Northern Ireland (17/NI/0051).

### Procedures

Patients attended for a single study visit. They completed a number of asthma and cough-related PRO tools (ACQ-5, Mini-AQLQ, LCQ, Cough-specific Quality of Life Questionnaire (CQLQ), and visual analogue scales for cough severity and urge to cough). Patients also completed (in the following order), exhaled nitric oxide fraction (*F*_ENO_) measurement (NIOX; Aerocrine/Circassia Pharmaceuticals, Morrisville, NC, USA), lung function testing and a citric acid cough challenge test, and provided a blood sample for a peripheral blood eosinophil count. At the end of the visit, a cough monitor was attached (Leicester cough monitor (LCM)) for 24-h ambulatory cough monitoring at home. Patients were provided with a diary to document their time of going to sleep and time of wakening in the morning to determine the time period for “night-time” coughs. The LCM has previously been used to investigate cough in patients with asthma [12–14].

### Outcome measures

The study was exploratory in nature with the main outcome being the relationship of T2 inflammatory biomarkers with cough morbidity as measured by 24-h cough frequency. Patients were assigned to a T2 inflammatory composite biomarker group on the basis of *F*_ENO_ and peripheral blood eosinophil measurements at the time of study visit (see Statistical analysis).

Secondary outcomes included the relationship of asthma PROs with both objective measures of cough and cough PROs.

### Statistical analysis

Cut-points for *F*_ENO_ and peripheral blood eosinophil counts for group assignation were chosen as 20 ppb and 150 cells·µL^−1^, respectively, based on criteria published in GINA guidelines [1]. A patient was classified as either T2-low (*F*_ENO_ <20 ppb and peripheral blood eosinophils <150 cells·µL^−1^), T2-intermediate (*F*_ENO_ ≥20 ppb or peripheral blood eosinophils ≥150 cells·µL^−1^) or T2-high (*F*_ENO_ ≥20 ppb and peripheral blood eosinophils ≥150 cells·µL^−1^). One-way ANOVA was performed to compare differences in outcome measures between these patient groups.

A cough frequency of 5 coughs·h^−1^ was defined as the threshold for abnormal cough frequency, based on previous work which showed a normal mean cough frequency for healthy subjects of 2 coughs·h^−1^ with an upper 95% confidence interval of 5 coughs·h^−1^ [15]. To provide further clinical context we determined the proportion of asthmatic patients with cough frequency comparable to that typically observed in a chronic cough population where a cough frequency threshold of 10 coughs·h^−1^ was used as the lower threshold for inclusion into trials of novel anti-tussive therapies [16].

No prior data existed to allow a power calculation; however, based on similar studies, a sample size of 60 patients (40 severe asthma and 20 mild-to-moderate asthma) was selected [9, 17] while also considering the limited recruitment potential of only one recruitment site.

Data were analysed using SPSS version 25 (IBM, Armonk, NY, USA). Distribution testing was assessed using normality plots and Kolmogorov–Smirnov testing. Data is shown as mean with standard deviation or median (interquartile range) as appropriate. Between-group differences were assessed using independent t-tests or the Mann–Whitney U-test as appropriate. Bivariate correlations were assessed using Pearson's or Spearman's correlation coefficients as appropriate.

Cough reflex sensitivity and cough frequency data were log transformed prior to analysis.

## Results

Demographic and clinical characteristics of the 61 study participants (42 severe asthma and 19 mild-to-moderate asthma) are illustrated in table 1. No significant differences were observed between the two groups with regard to age or T2 biomarkers. Consistent with all severe asthma cohorts, patients with severe asthma were more likely to be female (69% *versus* 42%; p=0.046), with greater body mass index (29.9 *versus* 26.3 kg·m^−2^; p=0.033), worse lung function (forced expiratory volume in 1 s 74.5% *versus* 95.0% predicted; p<0.001), worse asthma control (ACQ-5 score 1.9 *versus* 0.6; p=0.002) and on higher doses of inhaled corticosteroids (beclometasone dipropionate-equivalent 2000 *versus* 400 µg; p<0.001) when compared with mild-to-moderate patients.

**TABLE 1:** Demographic information for study participants with severe and mild-to-moderate asthma (n=61)

	**Severe (n=42)**	**Mild-to-moderate (n=19)**	**p-value**
**Female**	29 (69)	8 (42)	0.046
**Age (years)**	55.5 (51.0–62.5)	54.0 (43.0–63.0)	0.383
**ACQ-5 score**	1.9 (1.0–2.9)	0.6 (0.0–1.4)	0.002
**AQLQ score**	4.7 (3.1–5.5)	5.9 (5.3–6.6)	0.001
**BMI (kg·m^−2^)**	29.9 (27.4–34.2)	26.3 (22.8–31.2)	0.033
**FEV_1_ (% pred)**	74.5 (63.8–88.3)	95.0 (83.0–106.0)	0.001
**FVC (% pred)**	86.5 (79.0–99.8)	97.0 (88.0–106.0)	0.02
**FEV_1_/FVC**	66.9 (61.9–74.6)	76.9 (70.0–80.0)	0.01
***F*_ENO_ (ppb)**	24.5 (16.5–55.5)	19 (13–34)	0.350
**Peripheral blood eosinophils (×10^9^ L^−1^)**	0.26 (0.13–0.26)	0.26 (0.14–0.33)	0.446
**Daily ICS dose (BDP-equivalent µg)**	2000 (1600–2000)	400 (0–1000)	<0.001
**Taking daily oral prednisolone^#^**	22 (52)	0 (0)	
**Daily oral prednisolone dose (mg)**	7.5 (5–10)		

Data are presented as n (%) or median (interquartile range), unless otherwise stated. ACQ-5: Asthma Control Questionnaire-5; AQLQ: Asthma Quality of Life Questionnaire; BMI: body mass index; FEV_1_: forced expiratory volume in 1 s; FVC: forced vital capacity; *F*_ENO_: exhaled nitric oxide fraction; ICS: inhaled corticosteroid; BDP: beclometasone dipropionate. ^#^: refers to a maintenance dose.

In patients with severe asthma, six (14.3%) were composite T2-low, 14 (33.3%) were T2-intermediate and 22 (52.4%) were T2-high. In patients with mild-to-moderate asthma, three (17.6%) were classed as T2-low, nine (52.9%) were classed as T2-intermediate and five (29.4%) were classed as T2-high.

**FIGURE 1:**
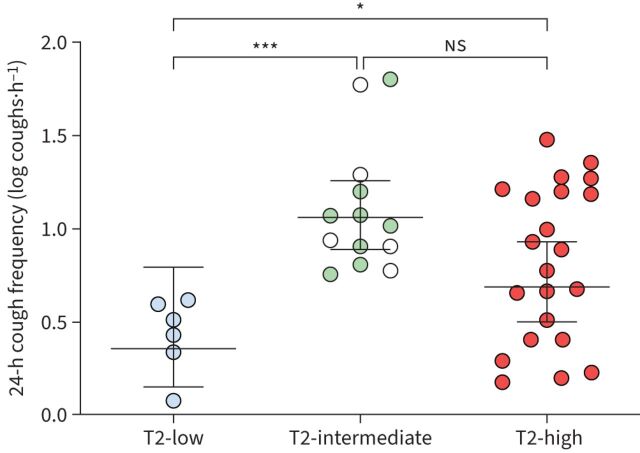
Distribution of cough frequency values in patients with severe asthma, separated by composite type 2 (T2) biomarker definitions. For T2-intermediate: green symbols indicate *F*_ENO_ <20 ppb and blood eosinophils ≥150 cells·µL^−1^ and open symbols indicate *F*_ENO_ ≥20 ppb and blood eosinophils <150 cells·µL^−1^. Data are presented as geometric means with 95% confidence intervals. ns: nonsignificant; *: p<0.05; ***: p<0.001.

Cough monitoring was completed in 58 patients (n=3 missing, due to equipment failure (n=2) and patient unwillingness to undergo ambulatory monitoring (n=1)). T2-low patients with severe asthma had significantly lower 24-h cough counts, cough frequency and daytime cough counts compared with T2-intermediate and T2-high patients (table 2 and figure 1). Additionally, cough frequencies associated with disordered coughing (≥5 and ≥10 coughs·h^−1^) were only present in those with raised T2 biomarkers and no patient in the T2-low group had an abnormal cough threshold.

**TABLE 2:** Values for cough measurement tools in patients with severe asthma grouped by composite biomarkers (n=42)

	**T2-low (n=6)**	**T2-intermediate (n=14^#^)**	**T2-high (n=22)**	**p-value**
**24-h cough count (n)**	64.0±1.6	305.6±2.2	157.4±2.6	0.002
**Daytime (awake) cough count (n)**	50.8±1.9	255.5±2.2	124.1±3.0	0.005
**Night-time (asleep) cough count (n)**	5.1±4.2	25.4±5.8	13.2±4.8	0.14
**24-h cough frequency (coughs·h^−1^)**	2.7±1.6	12.7±2.2	6.6±2.6	0.003
**Daytime (awake) cough frequency (coughs·h^−1^)**	3.1±1.8	18.0±2.2	9.1±2.8	0.002
**Night-time (asleep) cough frequency (coughs·h^−1^)**	1.7±1.9	3.6±4.3	2.2±3.4	0.48
**LCQ score**	18.7 (14.6–19.9)	14.2 (9.7–17.9)	15.2 (11.1–18.5)	0.21
**CQLQ score**	57.0 (34.5–62.3)	56.5 (46.8–73.5)	61.0 (53.0–68.3)	0.51
**VASc (mm)**	18.5 (5.8–27.3)	37.5 (19.0–68.8)	30.0 (11.8–48.5)	0.17
**VASu (mm)**	24.5 (12.5–32.3)	48.5 (20.5–68.8)	33.0 22.5–60.8)	0.31
***C*_2_ (M)**	0.25 (0.03–2.25)	0.125 (0.05–0.25)	0.125 (0.03–0.38)	0.82
***C*_5_ (M)**	0.50 (0.03–4.00)	0.25 (0.06–1.00)	0.125 (0.125–1.00)	0.94
***C*_max_ (M)**	0.50 (0.38–4.00)	4.0 (0.63–4.00)	1.0 (0.50–4.00)	0.66
***E*_max_ (n)**	17.0 (8.0–27.5)	24.0 (14.5–33.5)	18.0 (12.0–25.5)	0.37

Data are presented as mean±sd or median (interquartile range) unless otherwise stated. LCQ: Leicester Cough Questionnaire; CQLQ: Cough-specific Quality of Life Questionnaire; VASc: visual analogue scale for cough severity; VASu: visual analogue scale for urge to cough; *C*_2_: concentration of citric acid to elicit ≥2 coughs; *C*_5_: concentration of citric acid to elicit ≥5 coughs; *C*_max_: maximum concentration reached in cough challenge testing; *E*_max_: maximum number of coughs at any concentration during cough challenge testing. ^#^: cough monitoring failed for one patient so cough count and frequency values are only available for 13 patients.

Patients with mild-to-moderate asthma showed no differences between composite biomarker groups for any of the cough measures. There was also no evidence of any trends in cough frequency between the biomarker groups.

Patients with severe asthma had higher rates of cough than those with mild-to-moderate asthma in terms of total 24-h cough counts (geometric mean±sd 170.3±2.7 *versus* 60.8±4.1; p=0.002) and cough frequency (geometric mean±sd 7.1±2.7 *versus* 2.5±4.1 coughs·h^−1^; p=0.002). The frequency of coughing was higher during the day when compared with night for both patient groups (table 3). There was no significant difference in cough count between males and females in both patient groups. Cough frequency was also not influenced by any evidence of bronchiectasis or a recent positive sputum culture in patients with severe asthma (supplementary table E1).

**TABLE 3:** Values for cough measurement tools in patients with severe and mild-to-moderate asthma (n=61)

	**Severe (n=42)**	**Mild-to-moderate (n=19)**	**Effect size (95% CI)**	**p-value**
**24-h cough count (n)**	170.3±2.7	60.8±4.1	0.85 (0.26–1.44)	0.002
**Daytime (awake) cough count (n)**	136.9±2.9	55.7±4.0	0.72 (0.14–1.31)	0.01
**Night-time (asleep) cough count (n)**	14.1±5.2	4.5±4.5	0.73 (0.15–1.31)	0.02
**Cough frequency (coughs·h^−1^)**	7.1±2.7	2.5±4.1	0.87 (0.28–1.46)	0.002
**Daytime (awake) cough frequency (coughs·h^−1^)**	9.6±2.9	3.7±4.1	0.77 (0.19–1.35)	0.006
**Night-time (asleep) cough frequency (coughs·h^−1^)**	2.5±3.5	0.7±3.8	0.88 (0.29–1.46)	0.003
**LCQ score**	15.3 (11.1–18.5)	20 (17–20.5)	1.03 (0.43–1.62)	0.001
**CQLQ score**	60 (47.8–68.3)	37 (28–62)	0.78 (0.20–1.36)	0.01
**VASc (mm)**	27.0 (13.25–57.25)	8.0 (3.0–35.0)	0.69 (0.11–1.27)	0.003
**VASu (mm)**	33.5 (22–62.75)	10 (2–63)	0.47 (−0.10–1.04)	0.025
***C*_2_ (M)**	0.130 (1.35)	0.139 (1.38)	−0.19 (0.76–0.38)	0.86
***C*_5_ (M)**	0.290 (1.95)	0.413 (2.59)	−0.09 (0.66–0.47)	0.46
***C*_max_ (M)**	1.216 (16.4)	1.297 (19.81)	−0.08 (−0.64–0.49)	0.86
***E*_max_ (n)**	19 (13–28)	21 (9.25–26.5)	0.21 (−0.35–0.79)	0.59

Data presented as geometric mean±sd or median (interquartile range), unless otherwise stated. LCQ: Leicester Cough Questionnaire; CQLQ: Cough-specific Quality of Life Questionnaire; VASc: visual analogue scale for cough severity; VASu: visual analogue scale for urge to cough; *C*_2_: concentration of citric acid to elicit ≥2 coughs; *C*_5_: concentration of citric acid to elicit ≥5 coughs; *C*_max_: maximum concentration reached in cough challenge testing; *E*_max_: maximum number of coughs at any concentration during cough challenge testing.

In total, 25 (61%) patients with severe asthma and seven (41%) patients with mild-to-moderate asthma had an abnormally raised cough frequency of ≥5 coughs·h^−1^. Additionally, 15 (37%) patients with severe asthma and three (18%) patients with mild-to-moderate asthma had cough frequencies ≥10 coughs·h^−1^.

Scores for all cough PROs showed higher levels of cough morbidity in patients with severe asthma compared with those with mild-to-moderate asthma (table 3). In both patient groups there were wide ranges of scores observed for each measurement tool.

Cough challenge testing was completed in 39 patients with severe asthma and 16 patients with mild-to-moderate asthma (n=6 declined the test). No significant differences were observed between patients with severe asthma and mild-to-moderate asthma at the *C*_2_ and *C*_5_ end-points (concentration of citric acid to elicit ≥2 and ≥5 coughs, respectively) (table 2). Females with severe asthma had significantly lower *C*_2_ values compared with males (p<0.05) (supplementary figure E1), whereas no gender differences were observed in patients with mild-to-moderate asthma (supplementary figure E2).

Cough monitoring end-points were moderately correlated with ACQ-5 and Mini-AQLQ in patients with severe asthma, with stronger associations displayed in patients with mild-to-moderate asthma (table 4). Cough PROs and asthma PROs were more strongly correlated, in particular between LCQ and ACQ-5 (table 4).

**TABLE 4:** Correlation coefficients between asthma patient-reported outcomes and cough measurement end-points

	**Severe asthma (n=42)**		**Mild-to-moderate asthma (n=19)**	
	**ACQ-5**	**Mini-AQLQ**	**ACQ-5**	**Mini-AQLQ**
**24-h cough count (n)**	0.46**	-0.46**	0.50*	−0.58*
**24-h cough frequency (coughs·h^−1^)**	0.46**	−0.46**	0.51*	−0.58*
**LCQ**	−0.66**	0.66**	0.84**	0.78**
**CQLQ**	0.56**	−0.60**	0.33	−0.62**
**VASu**	0.48**	−0.45**	0.77**	−0.70**
**VASc**	0.56**	−0.55**	0.86**	−0.72**
***C*_2_**	−0.36*	0.24	−0.33	0.59*
***C*_5_**	−0.30	0.27	−0.26	0.37

Data are presented as Spearman's rank-order correlation coefficient. LCQ: Leicester Cough Questionnaire; CQLQ: Cough-specific Quality of Life Questionnaire; VASc: visual analogue scale for cough severity; VASu: visual analogue scale for urge to cough; *C*_2_: concentration of citric acid to elicit ≥2 coughs; *C*_5_: concentration of citric acid to elicit ≥5 coughs. *: p<0.05; **: p<0.01.

In patients with elevated cough frequencies above threshold values of 5 and 10 coughs·h^−1^, there were stronger relationships observed between cough monitoring measures and asthma PROs compared with those with “normal” cough frequencies (supplementary table E2). In patients with severe asthma, those with ACQ-5 ≥1.5 (*i.e.* uncontrolled asthma) had greater evidence of cough-related morbidity measured by cough PROs than those with ACQ-5 <1.5 (*i.e.* controlled asthma) (supplementary table E3).

Objective cough measures and cough PROs showed no correlation with *F*_ENO_ or peripheral blood eosinophils as individual markers of T2 inflammation in patients with severe and mild-to-moderate asthma (supplementary table E4). There were no associations between patterns of cough assessed by the cough monitoring and the measurements on any of the cough PROs.

## Discussion

This study has shown that cough morbidity, whether measured by objective cough monitoring or PRO tools, is common in asthma, is greater in subjects with severe disease, and is closely associated with PROs of asthma control and asthma quality of life. Furthermore, we have shown that while there is not a direct linear relationship between cough frequency and individual biomarkers of T2 inflammation, there appears to be a normal cough frequency and absence of cough-related morbidity in subjects with a composite T2-low biomarker profile (*i.e. F*_ENO_ <20 ppb and peripheral blood eosinophils <150 cells·µL^−1^). It is recognised that using a composite biomarker approach provides both better prognostication for severe exacerbation in asthma and response to corticosteroid treatment [18, 19]. In addition, the biomarker thresholds used in this study have been robust in predicting response to biologic therapies targeting the T2 cytokine axis [20, 21] and have been incorporated in the GINA statement on cut-points for identifying if T2 inflammation is present in patients with severe asthma. Based on these validated thresholds, our data suggest that cough morbidity appears to be minimal in those subjects with both mild-to-moderate and severe asthma, where biomarkers of T2 inflammation are low.

A long-standing view is that inflammation may induce neuronal sensitisation, which may be the underlying cause for cough in diseases such as asthma [22]. However, in previous work in severe asthma, it was suggested patient-reported cough morbidity (assessed by subjective cough-specific questionnaires) was not linearly related to T2 inflammation (assessed by the presence of sputum eosinophilia) [10]. A novel strength of our study was the use of objective cough monitoring; using this measure, the poor relationship between objectively measured cough frequency and both patient-reported cough morbidity and T2 biomarkers (surrogate measures of eosinophilic airways inflammation) has been confirmed. However, an important finding is that when objective cough measurements are used, patients with low T2 biomarkers have little evidence of increased cough frequency irrespective of asthma severity.

The number of patients with T2-low asthma in this study was small, but recent evidence testing a biomarker-directed strategy to optimise corticosteroid dose in severe asthma identified that the maximal prevalence of this phenotype in patients with severe asthma is ∼5% when corticosteroids are optimised and our numbers in this study are consistent with this figure [23]. The absence of significant cough burden in this biomarker-low group would suggest that suppression of T2 inflammation should be targeted initially in the management of cough in patients with asthma, but also suggests that when this is achieved, residual cough-related morbidity is likely to be low. A prospective placebo-controlled study in asthma patients with raised T2 inflammatory markers with effective anti-inflammatory treatment, with objectively measured cough monitoring, would be required to specifically address this issue. A study assessing the impact of anti-interleukin-5 therapy (mepolizumab) on objectively measured cough showed significant reductions in cough frequency after 6 months of therapy [24], supporting the concept that targeting eosinophilic inflammation in severe asthma has an impact on cough. While patients with severe asthma had higher cough frequency and higher scores on cough PROs compared with subjects with mild asthma, the absence of cough in those with low composite T2 biomarkers was consistent in both groups, as was the low frequency of night-time cough. Patients with severe asthma often have bronchiectasis and recurrent bacterial bronchitis, but this did not seem to relate to cough frequency, which may reflect their clinical stability at the time of study.

Notably, while asthma PROs such as the ACQ-5 and Mini-AQLQ do not specifically assess cough, we did note a correlation between measured cough frequency and these outcomes. This may be because cough is a significant contributor to the overall symptom burden associated with asthma, but may also mean that patients discriminate poorly between different symptoms (cough, wheeze, breathlessness, *etc.*) when responding to these questions, particularly when these symptoms are listed together in asthma PROs. For example, a question such as “On average, during the past week, how often were you woken by your asthma during the night?” in ACQ-5 is unlikely to specifically distinguish cough as the culprit symptom. Interestingly, cough-related questionnaires, which do ask cough-specific questions, were also significantly associated with asthma control, which supports previous work showing cough quality of life correlating well with asthma control measures [9, 25, 26]. Again, it is difficult to know if this is because of non-discrimination of individual respiratory symptoms in patients with asthma or whether it is identifying a specific cough burden, as the relationships between patient-reported cough measures and measured cough frequency were broadly similar across all comparisons. In addition, the relationship between asthma control and cough PROs when dichotomising patients around a cut-point in ACQ-5 score of 1.5 (where asthma is deemed to become “uncontrolled”) does detect a greater burden in cough-related morbidity.

Citric acid cough challenge testing was completed in 55 out of 61 patients and, importantly, was well tolerated in patients with severe asthma. There was no relationship between *C*_2_ or *C*_5_ thresholds and cough counts, which questions the utility of citric acid cough challenge in this patient population to identify those patients with high cough levels in asthma. Cough challenge testing has also not proved to be useful in detecting differences in cough morbidity between patients with asthma and healthy controls previously [27–29].

This study has also identified that cough morbidity, as measured by cough frequency, is potentially an important driver of asthma control and quality of life. However, this relationship appears to only become significant when a patient's cough becomes “abnormal”. This may be due to patients with higher cough counts being more aware of the symptom and therefore more responsive to symptom measurement tools. However, as the ACQ-5 does not directly measure cough (and the AQLQ only measures cough in a limited capacity) it is possible that patients with heightened cough do not discriminate between cough and other common asthma symptoms, which still results in a decrease in measurable asthma control.

A limitation of this study is that the number of patients was small; however, each of the patients had a very detailed assessment using subjective and objective measures of cough, and to the best of our knowledge this is the single biggest cohort of well-characterised severe asthma patients with cough monitoring. Another limitation is that patients only attended for a single study visit, where they completed all study measurements. While this is useful for providing an overview of a patient's condition at a specific point in time when clinically stable, it would also be useful to assess patients over a prolonged period of time with repeated measures, to assess any change in cough morbidity in line with other asthma measurements, particularly when asthma control deteriorates. Finally, it is worth noting that that there were a greater number of females in the severe asthma population that may have led to a more heightened cough reflex sensitivity and worse cough-related morbidity when compared with mild asthma, although severe asthma cohorts typically have a female preponderance [30, 31].

To conclude, a proportion of patients with severe asthma have significant cough burden; however, in patients with low biomarkers of T2 inflammation, cough morbidity is not seen. Adequate suppression of T2 inflammation would seem the logical initial step in managing cough in this population, although this will need to be confirmed in a larger series of patients. Based on the current data and given that effective therapies to suppress T2 inflammation are available, it seems unlikely that cough will be a separate treatable trait within a severe asthma population. Exploring how cough frequency and morbidity changes in response to suppression of T2 inflammatory biomarkers is important to better understand the role of cough within asthma.

## Supplementary material

10.1183/13993003.03205-2021.Supp1 **Please note:** supplementary material is not edited by the Editorial Office, and is uploaded as it has been supplied by the author.
Supplementary material ERJ-03205-2021.Supplement

## Shareable PDF

10.1183/13993003.03205-2021.Shareable1 This one-page PDF can be shared freely online.
Shareable PDF ERJ-03205-2021.Shareable
