# Supplementary material for: An observational study to determine the relationship between cough frequency and markers of inflammation in severe asthma
Source: Eur Respir J. 2022 Dec 8;60(6):2103205. doi: 10.1183/13993003.03205-2021 (PMC10436754; doi:10.1183/13993003.03205-2021)
Supplement: Supplementary file 1 [file ERJ-03205-2021.Supplement.pdf]

The relationship between cough frequency and markers of inflammation in severe asthma

Joshua Holmes, Lorcan PA McGarvey, Surinder Birring, Hannah Fletcher and Liam G Heaney

Online Data Supplement

<u>Selection Criteria</u>

Inclusion Criteria

<u>Severe asthma patients</u>

Severe asthmatic patients were recruited from a difficult asthma service (Belfast City Hospital). Patients were approached by their regular clinical team during a routine appointment visit and provided information about the research study.

Inclusion Criteria

1. Ability and willingness to comply with study procedures
2. Aged 18 to 75 years at the time of informed consent
3. Severe asthma (as defined by GINA step 4/5 classification of asthma severity) after a detailed systematic assessment
4. History of asthma treatment with high doses of ICS (≥1000 μg beclomethasone dipropionate daily, or equivalent) and an additional controller

<u>Mild/moderate patients</u>

Mild/moderate asthmatic patients were recruited from general respiratory clinics within the Belfast Health and Social Care Trust and from two participating general practice surgeries. Patients were sent study information sheets via their clinical team and invited to participate in the study. Any interested patients then contacted the study co-ordinator to express interest in study participation.

Inclusion Criteria

1. Previously received a physician diagnosis of asthma
2. Asthma defined as step 2/3 using the GINA asthma classification.
3. Aged 18 to 75 years inclusive
4. Have ability and willingness to comply with study procedures

Exclusion Criteria

Patients who met any of the following criteria were excluded from study entry:

1. Baseline FEV1 ≤50% of predicted or ≤ 1.0L
2. Asthma exacerbation within 28 days before the time of informed consent or during Screening
3. Major episode of infection requiring any of the following:
    a. Admission to hospital for ≥24 hours within the 28 days before the time of informed consent
    b. Treatment with intravenous antibiotics within the 28 days before the time of informed consent or during Screening
    c. Treatment with oral antibiotics within the 14 days before the time of informed consent or during Screening
4. Active tuberculosis (TB) requiring treatment within the 12 months before the time of informed consent (patients are also required to have no recurrence of symptoms in the 12 months following completion of TB treatment), or
5. Known history of severe clinically significant immunodeficiency, including, but not limited to, human immunodeficiency virus infection and/or currently receiving or have historically received intravenous Ig for treatment for immunodeficiency

    Note: Immunodeficiency encompasses a wide spectrum of human conditions and/or diseases. A relative IgG deficiency that is thought, but not proven, to be a feature of severe asthma would not be exclusionary for the study.

6. Diagnosis or history of malignancy, or current investigation for possible malignancy
7. Other clinically significant medical disease that is uncontrolled despite treatment or that is likely, in the opinion of the investigator, to require a change in therapy or affect the ability to participate in the study.
8. History of alcohol, drug, or chemical abuse that would impair or risk the patient's full participation in the study, in the opinion of the investigator
9. Current smoker or former smoker with a smoking history of >15 pack-years

    A current smoker is defined as someone who has smoked one or more

cigarettes per day (or marijuana or pipe or cigar) for ≥30 days within the 24 months before the time of informed consent and for whom cotinine testing is positive.

A former smoker is defined as someone who has smoked one or more cigarettes per day (or marijuana or pipe or cigar) for ≥30 days in his or her lifetime (as long as the 30-day total did not include the 24 months before the time of informed consent) and for whom cotinine testing is negative.

A pack-year is defined as the average number of packs per day times the number of years of smoking.

10. Initiation of or change in allergen immunotherapy within three months before the time of informed consent
11. Treatment with an investigational agent within 30 days of informed consent or 5 half-lives of the investigational agent, whichever is longer
12. Female patients who are pregnant or lactating

Table E1. High resolution CT scan and sputum growth analysis for patients with severe asthma

Patient No.	CT scan result	Sputum culture	Total coughs	Group	Coughs per hour
**A33**	No evidence of bronchiectasis	None within 5 years	1532.0	3	63.8
**B01**	No evidence of bronchiectasis	No significant growth	1439	3	60
**A05**	CT not clinically indicated	None	726	4	30.3
**A21**	No evidence of bronchiectasis	No significant growth	540	3	22.5
**A11**	CT not clinically indicated	None	473	4	19.7
**A04**	No evidence of bronchiectasis	Yeasts (abnormal)<br>No significant growth<br>Haemophilus influenzae (Abnormal)<br>Haemophilus influenzae (Abnormal)	460	2	19.2
**A10**	CT not clinically indicated	No significant growth	443	4	18.5
**A24**	Mild bronchiectasis	Haemophilus influenzae (Abnormal)	386	1	16.1
**A03**	CT not clinically indicated	No significant growth<br>Moraxella catarrhalis (Abnormal)	377	2	15.7
**A06**	No evidence of bronchiectasis	Haemophilus influenzae (Abnormal)	376	2	15.7
**A18**	Mild bronchiectasis in the upper lobes	Moraxella catarrhalis (Abnormal)	374	1	15.6
**A31**	No evidence of bronchiectasis	Yeats (abnormal)<br>No significant growth	356.0	3	14.8
**A17**	CT not clinically indicated	Yeats (abnormal)<br>No significant growth	288	4	12
**A16**	No evidence of bronchiectasis	No significant growth	282	3	11.8
**C02**	CT not clinically indicated	None within 5 years	250	4	10.4
**A29**	CT not clinically indicated	Stenotrophomonas maltophilia (Abnormal)	237	4	9.9
**A30**	No evidence of bronchiectasis	No significant growth	214	3	8.9
**A08**	CT not clinically indicated	None	198	4	8.3

Patient No.	CT scan result	Sputum culture	Total coughs	Group	Coughs per hour
**A07**	No evidence of bronchiectasis	No significant growth	195	3	8.1
**A19**	CT not clinically indicated	None	195	4	8.1
**A22**	CT not clinically indicated	Yeasts (abnormal)<br>No significant growth<br>Haemophilus influenzae (Abnormal)<br>Haemophilus influenzae (Abnormal)	181	2	7.5
**A12**	CT not clinically indicated	No significant growth	154	4	6.4
**A20**	CT not clinically indicated	Haemophilus influenzae (Abnormal)	145	2	6
**A15**	Modest bronchiectatic changes are found within all lung segments	No significant growth<br>Moraxella catarrhalis (Abnormal)	142	1	5.9
**A13**	No evidence of bronchiectasis	Haemophilus influenzae (Abnormal)	140	2	5.8
**A09**	CT not clinically indicated	Moraxella catarrhalis (Abnormal)	114	2	4.8
**C03**	No evidence of bronchiectasis	Yeats (abnormal)<br>No significant growth	113	3	4.7
**A02**	No evidence of bronchiectasis	No significant growth	111	3	4.6
**A23**	No evidence of bronchiectasis	None within 5 years	100	3	4.2
**C18**	No evidence of bronchiectasis	Stenotrophomonas maltophilia (Abnormal)	97	2	4
**A14**	CT not clinically indicated	No significant growth	76	4	3.2
**B03**	No evidence of bronchiectasis	None	76	3	3.2
**B04**	No evidence of bronchiectasis	No significant growth	64	3	2.7
**A25**	No evidence of bronchiectasis	None	62	3	2.6
**A27**	No evidence of bronchiectasis	Yeasts (abnormal)<br>No significant growth<br>Haemophilus influenzae (Abnormal)<br>Haemophilus influenzae (Abnormal)<br>Haemophilus influenzae (Abnormal)	62	2	2.6
**B02**	CT not clinically indicated	No significant growth	52	4	2.2
**A01**	Background mild cylindrical bronchiectasis	Haemophilus influenzae (Abnormal)	48	1	2
**C05**	CT not clinically indicated	No significant growth<br>Moraxella catarrhalis (Abnormal)	41	2	1.7

Patient No.	CT scan result	Sputum culture	Total coughs	Group	Coughs per hour
**A34**	CT not clinically indicated	Stenotrophomonas maltophilia (Abnormal)	39.0	2	1.6
**A26**	CT not clinically indicated	Haemophilus influenzae (Abnormal)	36	2	1.5
**A28**	Features are consistent with early bronchiectatic change	Moraxella catarrhalis (Abnormal)	28	1	1.2
**A32**	Mild bronchiectasis	No significant growth	N/A	1	N/A

Groups described as follows; 1: Evidence of bronchiectasis on HRCT, 2: No evidence of bronchiectasis on HRCT but with positive sputum culture for bacteria within prior 12 months, 3: No evidence of bronchiectasis on HRCT and no positive sputum culture for bacteria with prior 12 months, 4: HRCT scan not indicated as no clinical evidence to suggest bronchiectasis or bacterial infection

There were no differences in cough frequency bewteen the variouos groups described above

Table E2. Relationship between cough measurements and asthma patient reported outcomes when separated by cough frequency thresholds of 5 coughs/h and 10 coughs /hour

Cough measurement endpoint	ACQ-5				Mini AQLQ			
	**< 5 c/h** (N = 16)	**≥ 5 c/h** (N = 25)	**< 10 c/h** (N = 26)	**≥ 10 c/h** (N = 15)	**< 5 c/h** (N = 16)	**≥ 5 c/h** (N = 25)	**< 10 c/h** (N = 26)	**≥ 10 c/h** (N = 15)
**24-hour cough count**	0·42	0·65**	0·09	0·77**	-0·30	-0·70**	--0·08	-0·77
**24-hour cough frequency**	0·42	0·65**	0·09	0·78**	-0·30	-0·70	-0·08	-0·77**
**LCQ**	-0·36	-0·78**	-0·36	-0·86**	0·32	0·79**	0·42*	0·83**
**CQLQ**	-0·06	0·79**	0·22	0·76**	0·04	-0·84**	-0·25	-0·72**
**VASc (mm)**	0·14	0·55**	0·25	0·38	-0·17	-0·52**	-0·26	-0·26
**VASu (mm)**	0·32	0·63**	0·40*	0·46	-0·41	-0·59**	-0·43*	-0·30
**C_2 (M)**	-0·06	-0·58**	-0·24	-0·47	0·06	0·41*	0·08	0·46
**C_5 (M)**	-0·06	-0·48*	-0·17	-0·46	0·15	0·41*	0·09	0·53*

Data presented as Spearman's rank-order correlation co-efficient·
*** indicates p value < 0·05, ** indicates p value < 0·01**

Table E3: Median values for the Cough PROs in patients with severe asthma separated by ACQ-5 score cut point

	**ACQ-5 < 1·5 (n=15)**	**ACQ-5 ≥ 1·5 (n=27)**	***P value***
**Median LCQ score**	18·3 [16·2-20·2]	13·2 [9·5-16·7]	*0·001*
**Median CQLQ score**	50 [42-61]	62 [56-72]	*0·009*
**Median VASc (mm)**	20 [5 – 37]	39 [20 – 62]	0·03
**Median VASu (mm)**	22 [7 – 34]	49 [25 – 68]	0·02
**Data presented as median values [interquartile range]**			

Table E4. Correlation coefficients between cough measurements and individual T2 biomarkers

	Severe (n=41)		Mild / moderate (n=17)	
	FeNO	Blood eosinophils x10^9 cells /L	FeNO	Blood eosinophils x10^9 cells /L
**Total coughs (24 hours)**	0.07	0.19	-0.39	0.38
**Awake cough total**	0.09	0.16	-0.42	0.37
**Night-time cough total**	0.05	0.18	-0.34	0.31
**Coughs per hour**	0.06	0.19	-0.39	0.36
**Awake coughs per hour**	0.09	0.22	-0.40	0.38
**Night-time coughs per hour**	0.03	0.16	-0.33	0.29
**LCQ**	- 0.11	-0.09	0.39	-0.15
**CQLQ**	0.28	0.07	-0.31	0.20
**VASc**	0.13	0.01	-0.41	0.16
**VASu**	0.13	0.01	-0.47*	0.35
**C_2 (M)**	-0.10	-0.09	0.34	-0.16
**C_5 (M)**	-0.06	0.00	0.09	-0.29
**Cmax (M)**	-0.09	-0.10	0.39	-0.05
**Emax**	-0.05	0.11	0.10	0.39

Data presented as Spearman's rank-order correlation co-efficient.
* indicates p value < 0.05

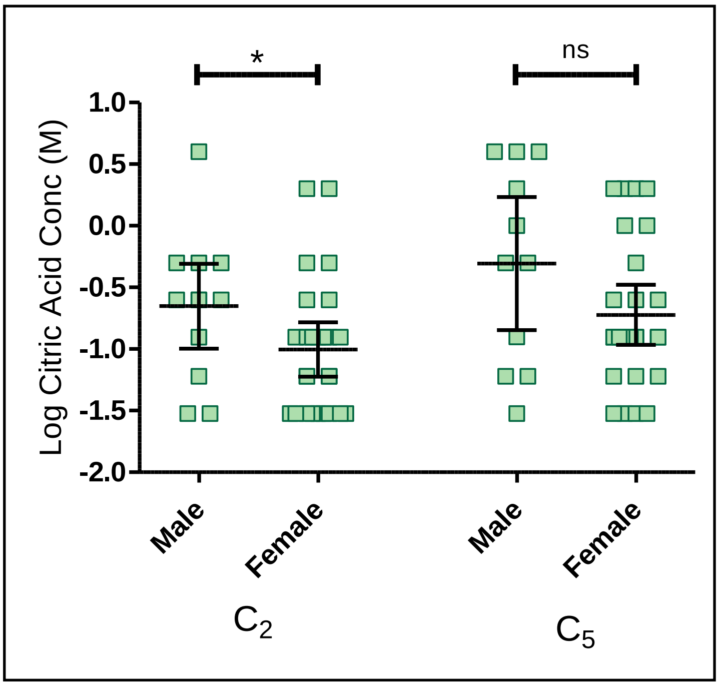

Figure E1: Log C2 and Log C5 values in severe asthma for males and females (with median and interquartile range) * indicates p value < 0.05, ns indicates p value not significant

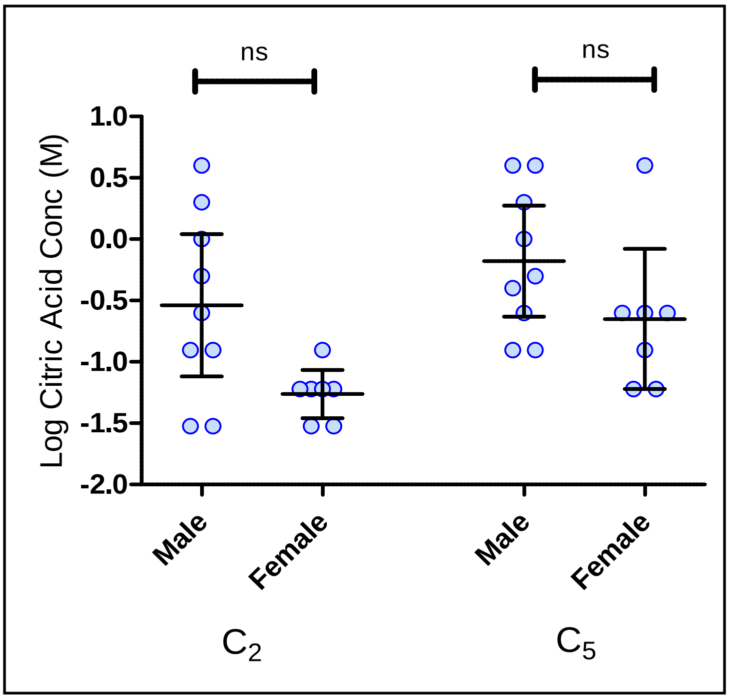

Figure E2: Log C2 and Log C5 values in mild/moderate asthma for males and females (geometric means and 95% confidence limits). ns indicates p value not significant
