# Supplementary material for: An observational study to determine the relationship between cough frequency and markers of inflammation in severe asthma
Source: Eur Respir J. 2022 Dec 8;60(6):2103205. doi: 10.1183/13993003.03205-2021 (PMC10436754; doi:10.1183/13993003.03205-2021)
Supplement: Supplementary file 2 [file ERJ-03205-2021.Shareable.pdf]

EUROPEAN RESPIRATORY JOURNAL
ORIGINAL RESEARCH ARTICLE
J. HOLMES ET AL.

SHAREABLE PDF

An observational study to determine the relationship between cough frequency and markers of inflammation in severe asthma

Joshua Holmes[1], Lorcan P.A. McGarvey[1], Surinder S. Birring[2], Hannah Fletcher[2] and Liam G. Heaney[1]

[1]Wellcome-Wolfson Institute for Experimental Medicine, Belfast, UK. [2]Centre for Human and Applied Physiological Sciences, School of Basic and Medical Biosciences, Faculty of Life Sciences and Medicine, King's College London, London, UK.

Corresponding author: Liam G. Heaney (l.heaney@qub.ac.uk)

Check for updates

Shareable abstract (@ERSpublications)
Although significant cough burden can be identified in patients with severe asthma, this may not be present in patients with low levels of T2 inflammatory biomarkers https://bit.ly/3yqSKVg

Cite this article as: Holmes J, McGarvey LPA, Birring SS, *et al*. An observational study to determine the relationship between cough frequency and markers of inflammation in severe asthma. *Eur Respir J* 2022; 60: 2103205 [DOI: 10.1183/13993003.03205-2021].

This single-page version can be shared freely online.

Copyright ©The authors 2022.

This version is distributed under the terms of the Creative Commons Attribution Licence 4.0.

Received: 20 Dec 2021
Accepted: 21 June 2022

Abstract

Background The relationship between objectively measured cough and type 2 (T2) biomarkers and other measures of asthma control and severity is poorly understood. The objective of this study was to assess the relationship between objective and subjective cough measurement tools and clinical biomarkers of asthma.

Methods Patients with severe asthma and mild-to-moderate asthma completed validated asthma and cough-related measurement tools (including ambulatory cough monitoring) and measurement of spirometry and T2 biomarkers (exhaled nitric oxide fraction (F_{ENO}) and peripheral blood eosinophil count). Patients were classified according to T2 status based on T2-low (F_{ENO} <20 ppb and peripheral blood eosinophils <150 cells·µL^{-1}), T2-intermediate (F_{ENO} ⩾20 ppb or peripheral blood eosinophils ⩾150 cells·µL^{-1}) or T2-high (F_{ENO} ⩾20 ppb and peripheral blood eosinophils ⩾150 cells·µL^{-1}).

Results 61 patients completed the study measurements (42 severe asthma and 19 mild-to-moderate asthma). Patients with severe asthma had higher rates of cough than those with mild-to-moderate asthma in terms of total 24-h cough counts (geometric mean±SD 170.3±2.7 *versus* 60.8±4.1; p=0.002) and cough frequency (geometric mean±SD 7.1±2.7 *versus* 2.5±4.1 coughs·h^{-1}; p=0.002). T2-low patients with severe asthma had significantly lower 24-h cough frequency compared with T2-intermediate and T2-high patients.

Conclusions In patients with low biomarkers of T2 inflammation, cough frequency measurements were not elevated, suggesting that the mechanism for cough in asthma is underlying T2 eosinophilic inflammation and the logical first step for treating cough in asthma may be to achieve adequate suppression of T2 inflammation with currently available therapies.
